# Qualitative discrimination of Chinese dianhong black tea grades based on a handheld spectroscopy system coupled with chemometrics

**DOI:** 10.1002/fsn3.1489

**Published:** 2020-02-28

**Authors:** Jing Huang, Guangxin Ren, Yemei Sun, Shanshan Jin, Luqing Li, Yujie Wang, Jingming Ning, Zhengzhu Zhang

**Affiliations:** ^1^ State Key Laboratory of Tea Plant Biology and Utilization Anhui Agricultural University Hefei China

**Keywords:** Chinese dianhong black tea, Grade discrimination, handheld near‐infrared spectroscopy, Partial least‐squares discriminant analysis, support vector machine

## Abstract

The evaluation of Chinese dianhong black tea (CDBT) grades was an important indicator to ensure its quality. A handheld spectroscopy system combined with chemometrics was utilized to assess CDBT from eight grades. Both variables selection methods, namely genetic algorithm (GA) and successive projections algorithm (SPA), were employed to acquire the feature variables of each sample spectrum. A partial least‐squares discriminant analysis (PLS‐DA) and support vector machine (SVM) algorithms were applied for the establishment of the grading discrimination models based on near‐infrared spectroscopy (NIRS). Comparisons of the portable and benchtop NIRS systems were implemented to obtain the optimal discriminant models. Experimental results showed that GA‐SVM models by the handheld sensors yielded the best predictive performance with the correct discriminant rate (CDR) of 98.75% and 100% in the training set and prediction set, respectively. This study demonstrated that the handheld system combined with a suitable chemometric and feature information selection method could successfully be used for the rapid and efficient discrimination of CDBT rankings. It was promising to establish a specific economical portable NIRS sensor for in situ quality assurance of CDBT grades.

## Graphical abstract

The handheld near‐infrared spectroscopy (NIRS) system combined with a suitable chemometric and feature information selection method could successfully be used for the rapid and efficient discrimination of Chinese dianhong black tea (CDBT) grades. It was promising to establish a specific economical portable NIRS sensor for in situ quality assurance of CDBT grades.

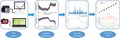

## 1 INTRODUCTION

Tea is one of the healthiest beverages consumed across the world (Ren, Fan, He, Li, & Tang, 2020; Zareef et al., 2019). The tea infusion is rich in various nutrition for human healthy, such as tea polyphenols, caffeine, free amino acids and vitamin, which has the effect of anti‐oxidation, hypotensive, anti‐carcinogenic, and protection against cardiovascular disease (Jiang, Yu, et al., 2019; Zhang, Santos, et al., 2019; Zhou, Zhao, et al., 2019). Chinese dianhong black tea (CDBT) is cultivated as a representative and influential black tea production from *Camellia sinensis* in China and has become one of the famed international black tea brands. CDBT is as a large‐leaf variety (var. assamica) classified into different grades in accordance with the variable in black tea quality that determined by growing condition, harvesting season and process technology. In general, ordinary consumers hold the traditional views that a high price represents an excellent quality of goods in the market (Zhu et al., 2019). Hence, the price of CDBT is nearly the only pathway for normal consumers to evaluate tea quality and discriminate its grades. Besides, the adulteration of CDBT and serving inferior products as superior ones are quite widespread in the tea trade which damages consumer rights and the far‐reaching tea culture (Li, Wei, Ning, & Zhang, 2015; Xu, Wang, & Gu, 2019). It is urgent to build a reliable and efficient method for discriminating the quality grades of CDBT (Pang et al., 2012).

During the last few decades, the quality assessment of CDBT is decisively judged by the sensory tests of the experienced tea tasters (Pan, Sun, Li, Deng, & Zhang, 2019). The skilled experts grade the tea samples on a scale, separately for appearance, taste, and aroma, which lacks reproducibility and impartiality due to the tasters' physical or physiological factors (Ren et al., 2013). The contents of main biochemical constituents (e.g., catechins, tea pigments, and amino acids) from different tea rankings simultaneously affect its quality parameters. Currently, the sensory evaluation combined with chemical composition analysis is considered as a more accurate the assessment scheme of CDBT grades. Conventional methods of chemical analysis have been used to determine the main chemical components of tea, such as gas chromatography (GC) (Wu et al., 2019), high‐performance liquid chromatography (HPLC) (Zhang, Jing, et al., 2019; Zhou, Sun, et al., 2019), and colorimetric measurements (Wang et al., 2019). However, all of the above mentioned methods are time‐consuming and high‐cost techniques. The traditional means are unable to achieve the rapid assessment of CDBT quality rankings. So far, the fast detection approaches for tea quality grades mainly involve nanotechnology (Zhu et al., 2019), colorimetric sensor array‐based artificial olfactory (Jiang, Xu, & Chen, 2019; Li, Xie, et al., 2017), and near‐infrared spectroscopy (NIRS) coupling with chemometric methods (Fu, Xu, Yu, Ye, & Cui, 2013; Ikeda, Kanaya, Yonetani, Kobayashi, & Fukusaki, 2007). While, the nanotechnology and colorimetric sensor array based on the reaction of toxic chemical reagents are not mature enough from practical applications. NIRS has proven to be a powerful analysis tool applied widely for qualitative identification in the agricultural and food industries (Deng et al., 2020). NIRS technique evaluates characteristic information through the analysis of the molecular bonds in the NIRS band (e.g., C‐H, N‐H, O‐H, and S‐H), which are the primary structural components of organic molecules (Ren et al., 2013). At present, extensive research on using NIRS technique in tea is reported. Only two studies have recorded the application of NIRS method to the discrimination of tea grades (Fu et al., 2013; Ikeda et al., 2007). Both published papers are specific to green tea, and that spectral feature variable screening algorithms are not applied and discussed nor were the comparisons of both benchtop and handheld NIRS instruments used to predigest the models. Besides, no study has performed an experiment to distinguish black tea of eight grades by using the self‐developed system because of higher rankings denote, smaller differences, and thus greater difficulty in tea identification (Zhu et al., 2019).

**Figure 1:**
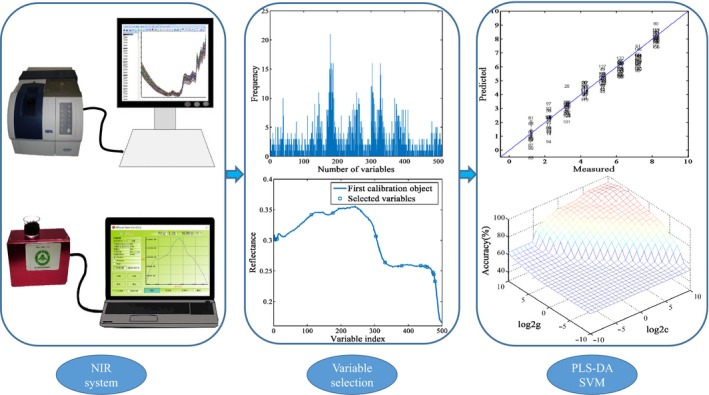
Schematic representation of both benchtop and handheld NIRS systems combined with chemometric algorithms. NIRS, near‐infrared spectroscopy; PLS‐DA, partial least‐squares discriminant analysis; RMSECV, root mean square error of cross‐validation; SVM, support vector machine

In this work, the main goal is to design an identification model to discriminate CDBT grades by utilizing a low‐cost handheld near‐infrared sensor combined with chemometric methods (Figure 1). A comparison for the handheld and benchtop devices were executed to verify the feasibility of establishing a reliable and inexpensive discriminant model. PLS‐DA and support vector machine (SVM) combined with genetic algorithm (GA) and successive projections algorithm (SPA) methods were applied comparatively in order to select the optimal recognition model and provide an advisable method for the portable NIRS system.

## 2 MATERIALS AND METHODS

### 2.1 Sample preparation

A total of 240 CDBT samples were collected from Yunnan province in southwest China. All samples processed into eight quality grades were harvested in 2018 within the tea planting base of Yunnan Dianhong Group Co. Ltd. The number of samples from each grade contained 30. Every sample was ground for 10 s by a high‐speed grinder (Beijing ever briGht medical treatment instrument co., Ltd, model FW100). The ground tea (50 g) was sieved with through a 40 mesh and then used for spectra acquisition, and the sieved samples were packed into kraft paper bags and stored in an airtight and dark place for further analysis.

### 2.2 Spectra acquisition

The data were acquired by Bruker MPA Fourier transform (FT) near‐infrared spectrometer (Bruker Optik GmbH) and a handheld system by independent development, respectively. The benchtop NIRS instrument with an integrating sphere was used to record the diffuse reflectance spectra of the samples between the wavelengths of 900 and 1,700 nm at 8 cm^−1^ resolution by 32 scans. The data were measured by 3.86 cm^−1^ interval, which resulted in 1,354 variables in each spectrum. For each sample, 3.0 ± 0.1 g of tea powder was packed into the quartz cell (35 mm diameter), as is the standard procedure for the bulk density of materials. Every sample was measured three times after intercalated 120° cup rotations (Wang et al., 2013). The handheld near‐infrared spectrometer recorded spectra in diffuse reflection mode in the range between 11,100 cm^−1^ and 5,880 cm^−1^ (900–1,700 nm) with a resolution of 5.85 cm^−1^, was composed mainly of hardware and software systems. The hardware comprised the spectrometer, electric source, laptop, and the sample cup. The software included USB communication technology. The spectrometer with digital light procession and single‐phase Indium Gallium Arsenide (InGaAs) detector was produced by Texas Instruments. About 3.0 ± 0.1 g of each sample was put into a quartz cell. The spectra were measured consecutively three times with 32 scans, resulting in 512 variables. Performance differences between benchtop and handheld spectrometers were described in Table 1. The quartz cuvette was rotated manually between measurements. The mean spectra from each sample were used in the further analysis. The temperature was kept around 25°C, and the humidity was kept at a steady level in the laboratory.

**Table 1:** Performance differences between the benchtop and the handheld near‐infrared spectroscopy spectrometers

Parameters	Benchtop spectrometer	Handheld spectrometer
Optical instrument	Fourier transform	Digital micromirror
Wavelength range (nm)	900–1,700	900–1,700
Variables	1,354	512
Scan times	32	32
Resolution ratio (cm^−1^)	8	5.85
Detector	PbS	Single‐phase InGaAs
Size (cm)	58 × 38 × 26	12 × 8.5 × 5.4

### 2.3 Spectral data preprocessing

The raw spectra acquired from the NIR spectrometer were prone to be affected by the physical properties of the samples, background information, and noise interferences (Wang et al., 2017). Since the dry tea powders were composed of different particle sizes, and the scattering of light was high and variable (Huang, Li, Zhao, Huang, & Chen, 2015), the raw spectral data were subjected to spectral preprocessing to reduce the interference and enhance the contribution of the sample feature attribute before model building. Standard normal transformation (SNV) was selected as a superior method for the correction of light scatter in this study, which is a mathematical transformation means of the log (1/R) spectra used for removing slope variation and correcting for scattering effects (Ren et al., 2013). Each spectrum was corrected individually by two steps: first, centering the spectral values and, then second, scaling by the standard deviation calculated on individual spectral values (Barnes, Dhanoa, & Lister, 1989; Chen, Tan, Lin, & Wu, 2018).

### 2.4 Multivariate data analysis

Principal component analysis (PCA) is widely employed to identify and eliminate outliers, reduced the dimensionality of the existing data set, and extracted important information (Chen, Tan, & Lin, 2019; Li, Zhang, Zhao, Huang, & Wang, 2017). The analysis can simplify the data of raw spectra into several variables and eliminate collinearity and reduce the machine learning time while retaining the spectral information correlated with the black tea grades (Subbuthai, Periasamy, & Muruganand, 2012).

Partial least‐squares discriminant analysis (PLS‐DA) is widely applied as a simple, fast, relative good performance, and linear discrimination method for qualitative analysis (Costa, Uchida, Miguel, Duarte, & Lima, 2017). The methodology was implemented to explore what were the components or latent variables which better discriminate between different grades of samples from their NIR spectra (X matrix: 900–1,700 nm) according to their maximum covariance with a target class defined in a class pertinence variable (Y matrix: grades) (Bassbasi, De Luca, Ioele, Oussama, & Ragno, 2014; Genisheva et al., 2018). The performance of the PLS‐DA model was assessed in terms of the correlation coefficients of calibration (*R*_c_) and prediction (*R*_p_), the root mean square error of cross‐validation (RMSECV), and the root mean squared error of prediction (RMSEP). To obtain a better performance, in general, an excellent model should be allocated with the lower RMSECV, RMSEP, and the higher R (Ren et al., 2013).

Support vector machine (SVM) has proven to be a remarkable nonlinear pattern classification method used to establish a global model which is capable of dealing efficiently with high‐dimensional input vectors (Thissen, Pepers, Üstün, Melssen, & Buydens, 2004). SVM is based on VC dimension theory and structural risk minimization principle, which is devoted to improving the generalization ability (Wang et al., 2020). At present, SVM has been extensively utilized in practical application. In general, the algorithm is dependent on the optimal combination of two primary parameters (viz. penalty parameter and kernel function) to obtain satisfactory predicting results (Chen, Zhao, Fang, & Wang, 2007; Smola & Schölkopf, 2004). Penalty parameter (*c*) is employed to evaluate between minimizing the training error and model complexity. Kernel function parameter (*g*) defines the nonlinear mapping from input space to certain high‐dimensional feature space (Zhang, Liu, & Wang, 2008). Traditionally, the selected both parameters were based on radial basis function. Kernel functions had a significantly important function both in the theory and application of SVM (Xu, Jiang, Liu, He, & Chen, 2019).

### 2.5 Variables selection method

Two different variable selection algorithms were employed to extract characteristic variables information and reduce the complexity of the classification model in this study, namely, GA and SPA. GA is utilized as a heuristic search algorithm to implement an automated wavelength selection procedure for building multivariate calibration models based on partial least‐squares regression with self‐organization abilities and high robustness. The method aims to seek the optimum parameters from a population of candidate solutions by variable selection and select representative characteristic variables and improved the accuracy of the models while optimizing the outcomes (Ning et al., 2018; Xu et al., 2018). SPA is proposed as a flexible variable selection strategy for multivariate calibration (Fan et al., 2019). The technique is an advanced selective method designed to reduce the number of variables used for modeling, minimize collinearity from each wavelength number position (Liu & He, 2009), and improve the conditioning of multiple linear regression by minimizing collinearity effects in the calibration data set (Sun et al., 2019). The above optimization algorithms could remove the spectral regions with large noise and irrelative information and enhanced the predictive ability of modeling (Basati, Jamshidi, Rasekh, & Abbaspour‐Gilandeh, 2018; Li & Su, 2018).

### 2.6 Software

All the algorithms were implemented in Matlab R2014a (Mathworks) and SIMCA 14.1 software (Umetrics) under Windows 8.1 in data processing.

## 3 RESULTS AND DISCUSSION

### 3.1 Spectral features

**Figure 2:**
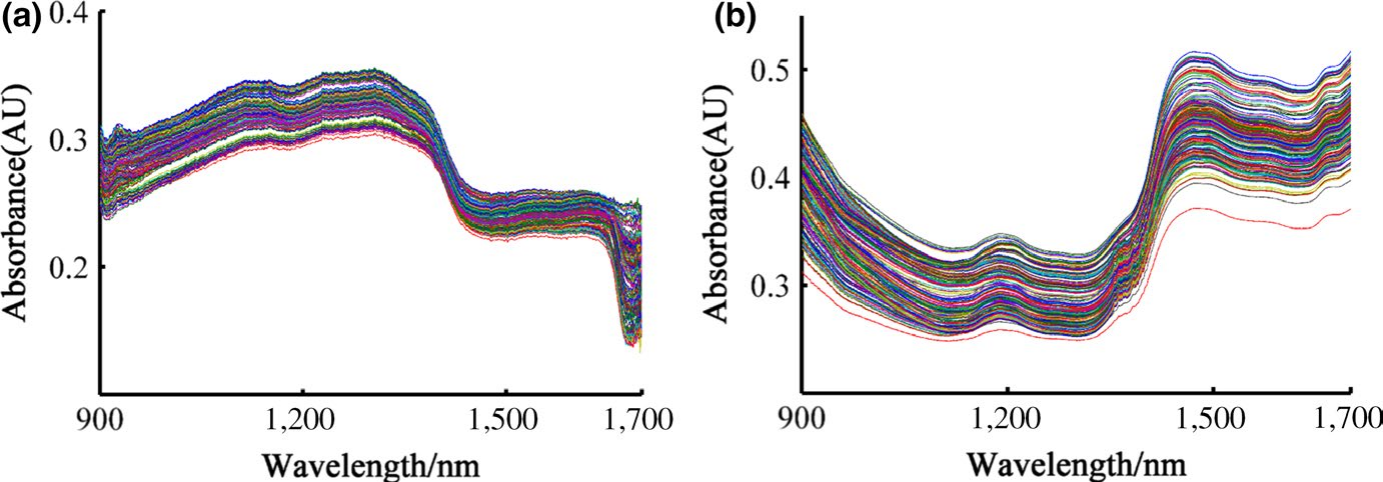
Raw spectra of all samples recorded with the handheld system (a) and benchtop spectrometer (b)

**Figure 3:**
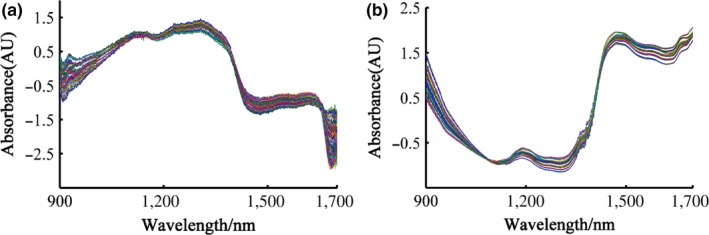
Spectral curves of the standard normal transformation method with the handheld system (a) and benchtop spectrometer (b)

Figure 2 shows the raw spectra of all the samples by using two different NIR instruments in absorbance. Original spectra collected on CDBT contains valuable information about the internal quality of the products. The stretching and bending vibrations of different functional groups (e.g., C‐H, O‐H, N‐H, and C‐O) associated with contents of water, protein, alkaloids, and other spectrally active components significantly influences the spectra (Li et al., 2019). Different groups have the otherness of absorption intensity and sites in the NIRS region. Seen from Figure 2, the spectrogram of tea samples from different grades was similar in trend, but slightly different in reflectance, which may be caused by the dissimilarities in the chemical component contents of tea from different grades. The spectral bands could be directly linked to the absorption features of the chemicals of interest (Bian et al., 2013). Water absorption was a dominated and characteristic peak at approximately 1,450 nm from bending vibrations of the O‐H bonds in the whole spectrum, and the other O‐H weak absorption bands were 1,200 nm. Besides, both disparate spectral detectors led to the absorbance differences. It was difficult to distinguish tea samples of different quality rankings from the raw spectral curves. The SNV pretreatment technology was utilized to the correction of the path length and the spectral intensity variation for further spectral analysis (Figure 3) (Xu et al., 2018).

### 3.2 Results of PCA

**Figure 4:**
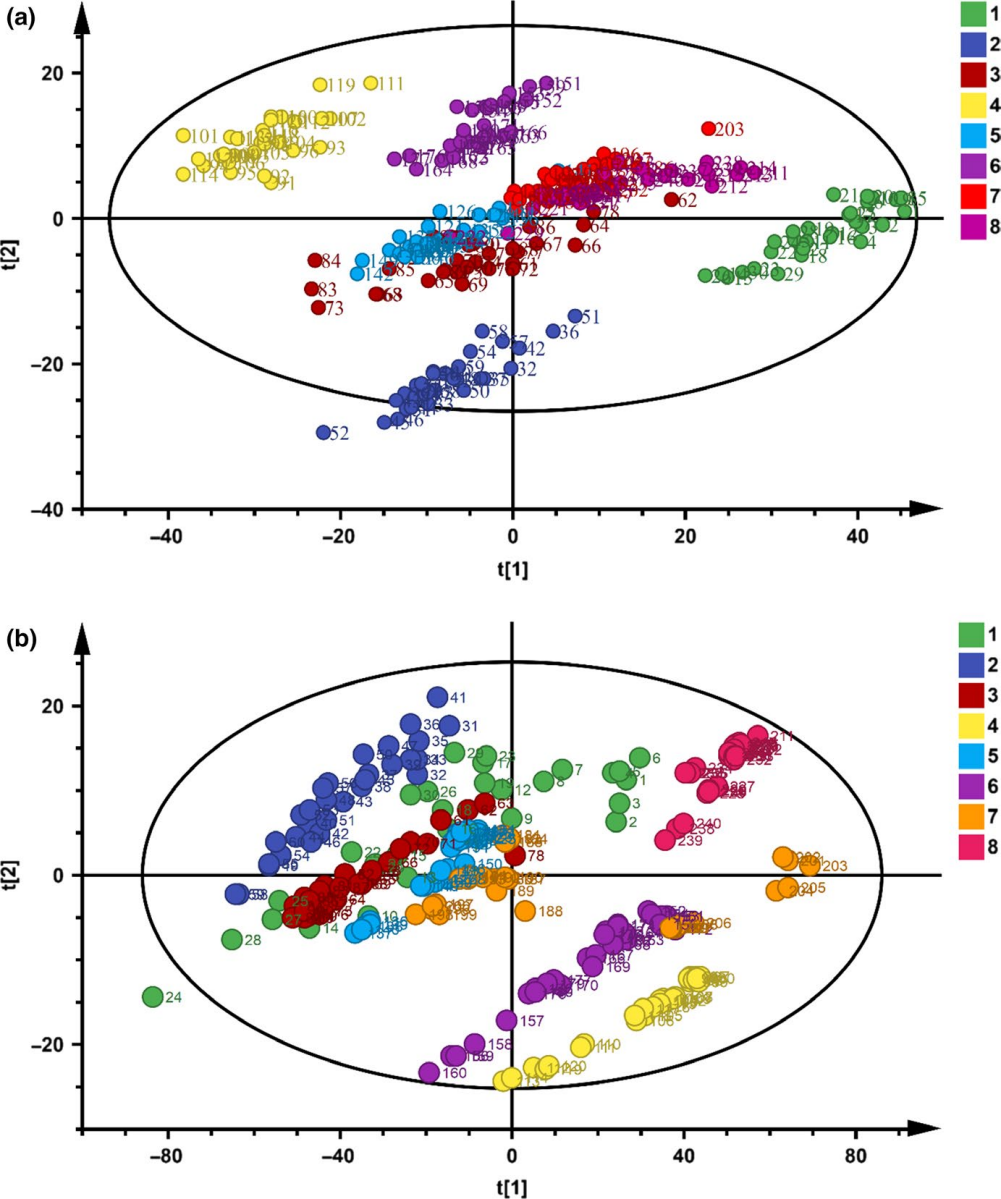
Principal component analysis score cluster plots for Chinese dianhong black tea of eight grades based on the handheld system (a) and benchtop spectrometer (b)

The pretreated spectra data of SNV could be reduced to several variables by PCA analysis. The analysis procedure investigated the accumulated variance contribution rate of the top two principal components (PCs) of the spectral data (Chen et al., 2019). Figure 4a shows the PCA classification results for CDBT of eight grades by the handheld system. The score for the first principal component (PC1) was 70.30%, which revealed an important information for the spectrum of the samples from each grade. The score of the second principal component (PC2) was 22.5%. The accumulated contribution rate of the first two PCs (PC1 and PC2) was 92.8%. The above data could represent the main spectral information of tea samples. From Figure 4a, the samples of four quality levels (namely, first, second, fourth, and sixth) appeared the significant spatial separation, and the spatial distribution of the samples from the other grades had overlapping. Figure 4b shows PCA score cluster plots for CDBT of eight grades based on the benchtop spectrometer. The first two principle components made up 97.39% of variance (PC1 = 89.70%, PC2 = 7.69%). The total contribution rate could present the primary spectrum information. The samples from every grade had their own cluster area. Clear boundaries in spatial distribution of the samples from partial grades (e.g., second, fourth, sixth, and eighth) were displayed from Figure 4b, and the scores of the remaining samples were still not completely separated. Therefore, PCA analysis was not appropriate for effective segregation of CDBT grades. The results need be further analyzed and identified by utilizing other modeling methods.

### 3.3 Optimal results of discriminant models via the two NIRS systems

All 240 samples obtained from eight grades were divided into a calibration subset and prediction subset. The former was employed to establish the calibration model and the latter to test the robustness of model. According to a 2/1 partition of data for calibration/prediction spectra, two of every three samples were selected into the calibration set. Thus, the calibration set contained spectra of 160 samples (viz. each grade of 20 samples), and the remaining spectra from 80 samples (viz. each grade of 10 samples) were assigned to the prediction set. Discriminant results for CDBT of different grades via the two NIR instruments (benchtop and handheld systems) are shown in Table 2. From the comparison of the both algorithms on benchtop NIRS data, the results of SVM models were a satisfactory strategy with the correct discriminant rate (CDR) of 100% for the training and testing sets. PLS‐DA modeling failed to give a completely effective performance based on the self‐developed system, and the CDR of the model only exceeded 80%. As can be seen from Table 2, the identification models obtained with the SVM approach resulted in calibration and prediction outcomes were superior to PLS‐DA. The possible reason was that the nonlinear SVM method could availably simplify the interaction between several variables well. For the above results, the identical excellent model performances were proposed from both desktop and portable devices. The handheld sensor was obviously used as a low‐cost and easy tool to achieve the high discriminant rates of CDBT grades with a potential for application industry‐wide.

**Table 2:** Discriminant results for CDBT of different grades via the two NIRS systems

Instrument type	Models	Parameters	CDR/(%)	
			Calibration set	Prediction set
Handheld	PLS‐DA	PCs = 10	88.75	81.25
	SVM	*c* = 0.7071, *g* = 0.5000	96.88	100.00
Benchtop	PLS‐DA	PCs = 10	98.13	100.00
	SVM	*c* = 2.8284, *g* = 0.7071	100.00	100.00

Abbrevaitions: CDBT, Chinese dianhong black tea; CDR, correct discriminant rate; NIRS, near‐infrared spectroscopy; PLS‐DA, partial least‐squares discriminant analysis; SVM, support vector machine.

### 3.4 Optimal results from different variables selection methods

In this study, two selection approaches applying for wavelength variable, which were GA and SPA, were used to select specific spectral bands and representative characteristic variables for establishing reliable reduced‐spectrum discrimination analysis models, achieving the rapid and accurate monitoring of CDBT from eight grades. However, the sampling strategies of the algorithms are both random. Hence, both methods were executed 100 times for the evaluation of reproducibility and stability in accordance with obtained statistical results. In PLS‐DA modeling, RMSECV, RMSEP, and R were used to appraise model performance. The number of variables selected and the number of optimal PCs were recorded. In addition, the maximum number of PCs was set as 15, and the optimal number of PCs achieved by a leave‐one‐out cross validation (CV) was then used for variables selection. GA was determined as the standard of the preferred variable for CV in terms of the global minimum of RMSECV. For running the GA, genetic iterations and mutation probability were set as 0.5 and 0.01, respectively.

**Figure 5:**
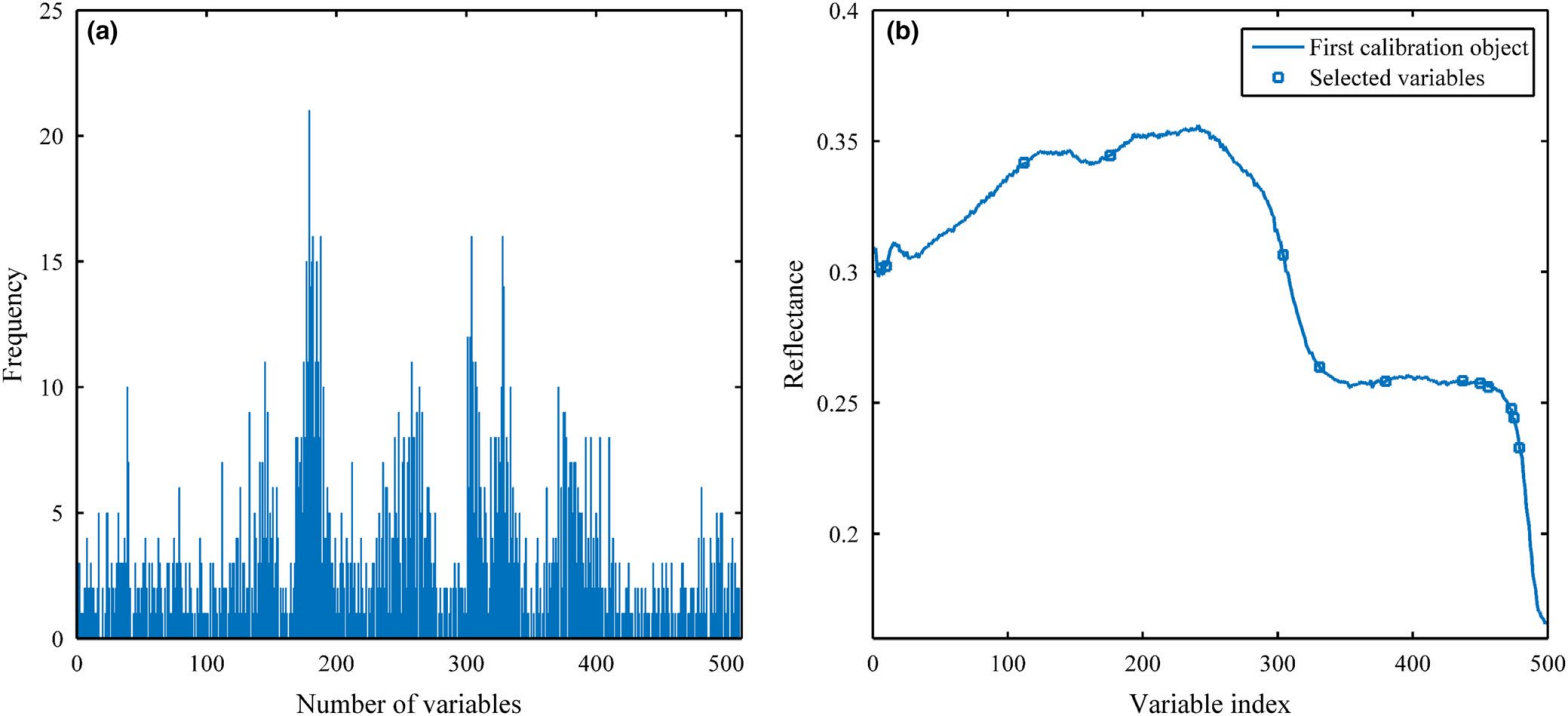
Results of selected variables by both variables selection methods. (a) Genetic algorithm; (b) successive projections algorithm

The qualitative statistical results of different variables selection methods (viz. GA and SPA) using the handheld sensor by 100 times execution are given in Table 3. The results show that the two variables screening methods have gratifying model performance both on calibration and on prediction set. Investigated from Figure 5, the selected variables by GA and SPA were fairly dispersed in full‐spectrum (including 512 variables) region. Compared with SPA and the full‐spectrum, the GA outperformed significantly with a smaller RMSECV and RMSEP, and a bigger R. Although, the variable sampling strategy of SPA algorithm collected fewer the number of variables (13 variables) from Table 3. The main reason for this phenomenon was that the variable extracting approach of each algorithm was different. SPA may ignore certain valid feature spectral information. Therefore, GA‐PLS‐DA identification model obtained better results with 90 variables and 7 PCs in validation process.

**Table 3:** Statistical results of different variables selection methods on the handheld NIRS sensor

Models	Variables	PCs	Calibration set		Prediction set	
			*R*_c_	RMSECV	*R*_p_	RMSEP
PLS‐DA	512	10	0.9904	0.3180	0.9870	0.3140
GA‐PLS‐DA	90	7	0.9955	0.2170	0.9906	0.3130
SPA‐PLS‐DA	13	13	0.9688	0.4966	0.9710	0.5450

Abbrevaitions: NIRS, near‐infrared spectroscopy; PC, principal component; PLS‐DA, partial least‐squares discriminant analysis; RMSECV, root mean square error of cross‐validation; RMSEP, root mean squared error of prediction.

### 3.5 Comparisons and discussion of optimal results from different modeling methods

Table 4 shows the predictive results of the optimal PLS‐DA and SVM models based on within 100 runs of both variables selection methods via the handheld sensor. As can be seen from Table 4, compared with the PLS‐DA and SVM models based on the SPA method, the other models based on the full‐spectrum and GA obtained better prediction results. Besides, compared with GA‐PLS‐DA, GA‐SVM, PLS‐DA, and SVM models, the GA‐SVM model acquired the best performance in the calibration and validation processes, and including the fewer wavelength variables. The corresponding CDR of the optimal GA‐SVM model reached 98.75% and 100% in the training set and prediction set, and two tea samples were merely misjudged as the other mixed grades. As for the causes from the handheld device with GA‐SVM algorithm achieving the best distinguishable performance, a brief discussions were expounded as follows:

**Table 4:** Results of the optimal PLS‐DA and SVM discrimination models based on different variables selection methods via the handheld NIRS sensor

Models	Parameters	Correct discriminant rate/%	
		Calibration set	Prediction set
PLS‐DA	PCs = 10	88.75	81.25
GA‐PLS‐DA	PCs = 7	96.25	90.00
SPA‐PLS‐DA	PCs = 13	67.50	72.50
SVM	*c* = 0.71 g = 0.50	96.88	100.00
GA‐SVM	*c* = 1 g = 4	98.75	100.00
SPA‐SVM	*c* = 8 g = 2.83	93.75	92.50

Abbrevaitions: GA, genetic algorithm; NIRS, near‐infrared spectroscopy; PC, principal component; PLS‐DA, partial least‐squares discriminant analysis; SPA, successive projections algorithm; SVM, support vector machine.

First, as for PLS‐DA and SVM models based on the full‐spectrum, the models were developed utilizing 512 spectral variables. However, in the full‐spectrum region, there existed multitudinous redundant variables that were unrelated to CDBT quality levels in tea grading. Thus, the predictive performance of the identification models would be likely weakened because of excessive useless information involving in modeling.

Second, the GA‐PLS‐DA, SPA‐PLS‐DA, GA‐SVM, and SPA‐SVM models were established by the application of the two informative spectral variables selection algorithms, respectively. The result of the GA‐SVM model was superior to that of PLS‐DA and SVM models using the full‐spectrum since the feature variables were extracted for calibrating the model. Moreover, compared with the PLS‐DA and SVM models, the corresponding SVM models obtained better results with the higher CDR of the calibration set and the prediction set in validation process. The main reason may explain the differences that the nonlinear pattern recognition approach of SVM possessed potential advantages for qualitative discrimination in contrast to that of the linear algorithm (i.e. PLS‐DA) in this study. The SVM method adopted a kernel function to map the data into a high‐dimensional space, where it could separate variable classes (Xu, Jiang, et al., 2019). The evaluation for CDBT quality ranking was a complex course involving internal physicochemical component transformation and composition metabolism, making recognition, and discrimination challenging.

Third, the results of classification models based on GA algorithm exhibited a higher CDR than that of the models using SPA method. Even though SPA combined with PLS‐DA or SVM reducing the signal‐to‐noise ratio could minimize information overlap and redundancy, and preferred variables (Diniz, Gomes, Pistonesi, Band, & de Araújo, 2014). The identification accuracy by applying the SPA algorithm was greatly affected with a low applicability. Actually, GA was more suitable for screening characteristic wavelengths related to tea grades in this paper. Therefore, the nonlinear GA‐SVM algorithm can achieve the best predictive results on the spectra via the developed system wherein low variables can obtain better generalization performance.

## 4 CONCLUSION

In this study, a developed handheld NIRS system combined with chemometric tools was presented for the rapid discrimination of CDBT grades. A comparison of the portable and benchtop devices was utilized to assess the qualitative model performances. The comparison results displayed that the SVM models using the above two apparatuses identified CDBT from eight grades with an accuracy rate of up to 100% between the wavelengths of 900 and 1,700 nm in the prediction set. Both algorithms (GA and SPA) for screening informative wavelength variables were proposed, and the two different machine learning methods (PLS‐DA and SVM) were employed to develop the discriminant models for the assessment of CDBT grades. From the results, the selected wavelength variable algorithms simplifying the model had a high discriminant rate on CDBT grades. The GA‐SVM methodology obtained a better performance by virtue of the predictive precision. This work demonstrated that the developed handheld NIRS system combined with an appropriate qualitative discrimination method has a high potential in the identification of CDBT grades, and it is promising to establish a specific economical portable NIRS sensor for in situ quality assurance of CDBT.

## CONFLICT OF INTEREST

The authors have declared no conflicts of interest for this article.

## ETHICAL APPROVAL

This study does not involve any human or animal testing.

## INFORMED CONSENT

Written informed consent was obtained from all study participants.
